# Activation of DNA-PK by Ionizing Radiation Is Mediated by Protein Phosphatase 6

**DOI:** 10.1371/journal.pone.0004395

**Published:** 2009-02-09

**Authors:** Jun Mi, Jaroslaw Dziegielewski, Elzbieta Bolesta, David L. Brautigan, James M. Larner

**Affiliations:** 1 Department of Radiation Oncology, University of Virginia Health System, Charlottesville, Virginia, United States of America; 2 Center for Cell Signaling, University of Virginia Health System, Charlottesville, Virginia, United States of America; Ordway Research Institute, United States of America

## Abstract

DNA-dependent protein kinase (DNA-PK) plays a critical role in DNA damage repair, especially in non-homologous end-joining repair of double-strand breaks such as those formed by ionizing radiation (IR) in the course of radiation therapy. Regulation of DNA-PK involves multisite phosphorylation but this is incompletely understood and little is known about protein phosphatases relative to DNA-PK. Mass spectrometry analysis revealed that DNA-PK interacts with the protein phosphatase-6 (PP6) SAPS subunit PP6R1. PP6 is a heterotrimeric enzyme that consists of a catalytic subunit, plus one of three PP6 SAPS regulatory subunits and one of three ankyrin repeat subunits. Endogenous PP6R1 co-immunoprecipitated DNA-PK, and IR enhanced the amount of complex and promoted its import into the nucleus. In addition, siRNA knockdown of either PP6R1 or PP6 significantly decreased IR activation of DNA-PK, suggesting that PP6 activates DNA-PK by association and dephosphorylation. Knockdown of other phosphatases PP5 or PP1γ1 and subunits PP6R3 or ARS-A did not reduce IR activation of DNA-PK, demonstrating specificity for PP6R1. Finally, siRNA knockdown of PP6R1 or PP6 but not other phosphatases increased the sensitivity of glioblastoma cells to radiation-induced cell death to a level similar to DNA-PK deficient cells. Our data demonstrate that PP6 associates with and activates DNA-PK in response to ionizing radiation. Therefore, the PP6/PP6R1 phosphatase is a potential molecular target for radiation sensitization by chemical inhibition.

## Introduction

DNA double-strand breaks (DSBs) arise from normal cellular processes such as V-D-J recombination and free radicals, as well as from exogenous sources, such as ionizing radiation or other forms of genotoxic stress. Homologous recombination (HR) and non-homologous end-joining (NHEJ) are the two major pathways for repair of DNA DSBs. NHEJ, which does not require the presence of a homologous template, is the predominant repair pathway for DSBs produced by ionizing radiation (IR). The DNA-dependent protein kinase (DNA-PK) plays a central role in regulating NHEJ, as evidenced by the hypersensitivity of DNA-PKcs (DNA-PK catalytic subunit)-/- mice to IR and the high levels of unrepaired DSBs observed in DNA-PKcs-/- mice after exposure to other forms of genotoxic agents [1].

DNA-PK has been classified on the basis of sequence analysis as a member of the phosphatidylinositol-3-kinase (PI-3-K)-related kinase (PIKK) super family [2], which includes the human ataxia telangiectasia mutated (ATM) and ATM-Rad3 related (ATR) proteins. These protein kinases regulate diverse processes, including genome surveillance and responses to cellular stress [3], [4]. DNA-PK is composed of a catalytic subunit (DNA-PKcs) and two Ku heterodimers, which act as regulatory subunits [5]. DNA-PKcs contains a DNA binding domain, a catalytic domain, and a Ku binding domain. NHEJ is initiated when two Ku heterodimers recognize and stably bind to broken DNA ends, where they serve to recruit two DNA-PKcs molecules to the damaged site [6]. Once bound to DNA, the kinase function of DNA-PKcs is activated. The two separate DNA-PK complexes interact with each other to bridge the two DNA ends through the N-terminal HEAT repeats of DNA-PKcs [7]. This interaction facilitates alignment of the two DNA ends for repair [8], [9].

Numerous studies have shown that DNA-PKcs undergoes a series of phosphorylations in response to DSBs at the clusters of ABCDE (six sites between Thr2609 and Thr2647) and PQR (five sites between residues 2023 and 2056) [10], as well as at additional conserved sites, including Thr3950 [11], [12]. Functional assays have revealed that phosphorylation at sites in these two clusters regulates DNA end-access to DNA end-processing factors and to other DNA repair pathways. Alanine substitution at all six sites of the ABCDE cluster virtually abolishes the ability of DNA-PK to function in NHEJ. However, mutating all five sites of PQR to alanine resulted in only a modest defect in NHEJ. The phosphorylation of ABCDE promotes end-processing, whereas the phosphorylation of PQR inhibits end-processing [6]. Thus, the ABCDE and PQR sites function reciprocally to regulate DNA end-access [12], [13]. Phosphorylation of the Thr3950 site is thought to be inhibitory, since mutants mimicking phosphorylation at this site lack kinase activity without a reduction in the affinity of the catalytic subunit for DNA-bound Ku. Moreover, impairing the kinase activity of DNA-PKcs or mutating the clusters of major phosphorylation sites does not block localization of DNA-PKcs to DSB sites, but lowers the rate of exchange between DNA-bound and free DNA-PKcs [12]–[17]. These observations suggest that autophosphorylation is required to destabilize the initial protein-DNA complex that, in turn, facilitates additional repair steps [8]. Ser/Thr phosphatases, such as PP5 and PP1γ1, are reported to be involved in the regulation of DNA-PK [18], [19]. The overexpression of PP5 decreased DNA-PKcs Thr2609 phosphorylation in HeLa cells, while purified PP1γ1 recovered the DNA-PK activity from autophosphorylated DNA-PKcs in an *in vitro* assay. However, little is known about which Ser/Thr phosphatases regulate DNA-PK activity through dephosphorylation of various sites in DNA-PKcs.

Protein phosphatase 6 (PP6) is a Ser/Thr protein phosphatase classified as a type 2A phosphatase family member based on its sequence homology to the catalytic subunit of protein phosphatase 2A (PP2A) [20] and its sensitivity to active site inhibitors such as okadaic acid, microcystin and calyculin A [21]. PP6 is functionally distinct from other type 2A phosphatases and conserved in evolution, because human PP6 rescues mutations of the homologous Sit4 in yeast [22]. PP6 plays a role in the regulation of NFκB signaling [23]. The holoenzyme of PP6 is proposed to be a heterotrimer that consists of a catalytic subunit (PP6c), a SAPS (Sit4-Associated Protein) subunit plus an ankyrin repeat subunit (ARS). The human SAPS, named as PP6R1, PP6R2 and PP6R3, are more divergent in sequence than PP6 and are widely distributed in multiple tissues [23]. Recent studies show that siRNA knockdown of PP6R1, but not PP6R3, enhances degradation of endogenous IκBε in response to tumor necrosis factor-α (TNF-α) [24]. These results suggest that one function of the SAPS-like subunit PP6R1 is to target PP6 to specific substrates such as IκBε.

In this study, we show that DNA-PKcs associates with PP6R1, that this binding increases after IR, and that depletion of PP6/PP6R1 reduces IR activation of DNA-PKcs and increases the radiosensitivity of glioblastoma cells. These observations suggest that PP6 with a PP6R1 subunit is an important regulator of DNA-PK activity and function in cells.

## Materials and Methods

### Cell lines and reagents

DNA-PKcs-proficient (M059K) and DNA-PKcs-deficient (M059J) glioblastoma cells were maintained in DMEM/F12 media (Invitrogen, Carlsbad, CA) supplemented with 10% fetal bovine serum (FBS, Invitrogen), 0.05 mM non-essential amino acids (Invitrogen) and 0.5 mM sodium pyruvate (Invitrogen). All cells were maintained at 37°C with 5% CO_2_ and were in an exponential growth phase at the time of radiation. The following commercial antibodies were used: anti-DNA-PKcs pan mouse monoclonal, anti-Ku86 mouse monoclonal and anti-tubulin mouse monoclonal (Santa Cruz Biotechnology, Santa Cruz, CA); anti-DNA-PKcs Thr2609 phosphospecific rabbit polyclonal (Abcam, City, State); anti-RPA2 mouse monoclonal and β-actin mouse monoclonal (Sigma, St. Luis, MO). Anti-PP6 chicken polyclonal antibody, anti-PP6R1 chicken polyclonal antibody, and anti-ARSα rabbit polyclonal antibody were provided by the Brautigan lab. The DNA-PK kinase assay kit was obtained from Roche. All other reagents were purchased from Sigma.

### Radiation treatment

Cells in culture were irradiated with a superficial X-ray machine at a dose rate of 1.48 Gy per minute. During irradiation, the cultures were maintained in a container designed to mimic the conditions of the cell culture incubator (5% CO_2_ and 95% air at 37°C).

### Western blot

Whole cell extracts, fractionated extracts and immunoprecipitates were separated by SDS-PAGE and transferred to nitrocellulose membrane. Proteins of interest were detected with specific antibodies, followed with infrared dye 700 or 800-conjugated secondary antibodies. Blots were scanned using an Odyssey infrared imaging system (LI-COR), and proteins were quantitatively analyzed by the Odyssey software.

### siRNA knockdown

Exponentially growing M059K or M059J cells were transfected with specific siRNA (50 nM) against PP6c or PP6R1, as described previously [24], or against DNA-PKcs, PP5, ARSα, PP6R3 or PP1γ1, using LipofectAMINE RNAiMAX (Invitrogen) according to the manufacturer's instructions. A nonspecific siRNA (sequence: 5′-AAAUCUUCGAGACAUUCUGUU) was used as a control. All siRNA oligonucleotides were purchased from Dharmacon.

### Fluorescent immunostaining

Cells that grew in chamber slides were rinsed once with PBS at room temperature, fixed with 5% paraformaldehyde at room temperature for 15 minutes, rinsed twice again with PBS, and permeabilized with 0.3% Triton X-100/0.3% SDS in PBS for 10 minutes at room temperature. Cells were rinsed 3 times with PBS and incubated in 5% normal goat serum (MP Biomedical, City, State) in PBS blocking solution for 30 minutes at room temperature. Mouse anti-DNA-PKcs or anti-phospho-DNA-PKcs Thr2609 monoclonal and chicken anti-PP6R1 polyclonal antibodies were diluted 1∶100 in 5% goat serum PBS and applied to the chamber slides for 2 hours at room temperature or overnight at 4°C. Cells were rinsed 3 times with PBS for 5 minutes each before staining with the appropriate secondary antibodies, including fluorescein isothiocyanate (FITC)-conjugated goat anti-rabbit and Texas red-conjugated goat anti-mouse, and diluted 1∶400 in 5% goat serum PBS for 1 hour at room temperature. Chamber slides were rinsed 5 times again with PBS as described above and mounted with 10 µL of Vecta-shield mounting medium containing 200 ng/mL DAPI (Vector Laboratories, Burlingame, CA). Background staining was determined by preparing identical chamber slides without primary antibody. Images of fixed cells were captured with Openlab software using a Nikon fluorescence microscope (Microphoto-SA) equipped with a Nikon Plan Apo ×40 oil immersion objective, filter sets for FITC, Texas Red, and 4,6-diamidino-2-phenylindole fluorophores, and a Hamamatsu Orca C4742-95 digital camera. Raw data images were converted to 8-bit tiff images in Openlab.

### Cellular fractionation

Cells were collected in ice-cold PBS. The cell pellets were resuspended for 5 minutes in a permeabilization buffer consisting of 10 mM HEPES pH 7.4, 10 mM potassium acetate, 50 µg/mL Digitonin, 1 mM PMSF, 1 mM Na_3_VO_3_, and 1 µg/mL protease inhibitors (aprotinin, leupeptin and pepstatin). The supernatants were used as a cytoplasmic extract. The pellets were washed with permeabilization buffer two times and extracted with nuclear lysis buffer (0.5% Nonidet P-40, 150 mM NaCl, 10 mM sodium phosphate (pH 7.2), 2 mM EDTA, 50 mM sodium fluoride, 0.2 mM Na3VO3, 1 mM PMSF, and 1 µg/mL aprotinin). Insoluble material was removed by centrifugation, and the supernatant was used as a nuclear extract.

### Immunoprecipitation

Exponentially growing M059K or M059J cells were irradiated with 10 Gy IR, then harvested at indicated time points, and lysed in 1 mL of lysis buffer (0.5% (v/v) NP-40, 5 mM EDTA, 2 mM EGTA, 20 mM MOPS, 1 mM PMSF, 20 mM sodium pyrophosphate, 30 mM sodium fluoride, 40 mM β-glycerophosphatase, 1 mM Na_3_VO_3_, and protease inhibitors) with caspase inhibitor Z-VAD-FMK. Aliquots of 1 mg total protein were mixed with 4 µg of monoclonal anti-DNA-PKcs antibody at 4°C overnight. The bound proteins were recovered by binding to 25 µL of protein-A agarose (Sigma). The samples were separated by 7.5% SDS-PAGE gel electrophoresis, then transferred to nitrocellulose overnight and analyzed by Western blot.

### DNA-PK kinase assay

Exponentially growing M059K or M059J cells were treated with 10 Gy IR. After 30 minutes, the nuclear extracts were prepared in lysis buffer containing 0.42 M sodium chloride and 1.5 mM magnesium chloride. DNA-PK activity was analyzed using a DNA-PK activity assay kit (Roche) [13], [25] according to the manufacturer's instructions.

### Clonogenic assay

The method of the clonogenic assay was adapted from Franken, et al [26]. Briefly, the treated and untreated control cells were harvested and re-seeded in a 100 mm dish at an appropriate density to obtain approximately 50–100 colonies. Following 10–14 days incubation, cells were fixed and stained with crystal violet, and colonies containing at least 50 cells were scored.

### Pulse-field gel electrophoresis

siRNA-transfected cells were sham-treated or irradiated with 10 Gy IR on ice. After irradiation, cells were harvested immediately or following a 3 hour recovery at 37°C. Harvested cells were embedded in InCert agarose (BMA, Rockville, MD) plugs and lysed in 0.5M EDTA, (pH 8), 2% Sarcosyl, 1mg/ml Proteinase K solution for 48 hours at 50°C. PFGE was carried out using a CHEF Mapper system (Bio-Rad, Hercules, CA) for 74 hours in 0.8% agarose, 1x TBE buffer at 14°C, with 35 minute pulse time at the field strength of 2 V/cm and 106 degree included angle. *S. cerevisiae* and *S. pombe* chromosomes (Bio-Rad) were included as DNA size markers. Following electrophoresis, gels were stained with ethidium bromide, photographed and analyzed with Image J software (NIH, Bethesda, MD). The fraction activity released (FAR) was calculated as an amount of DNA entering the gel divided by the amount of DNA remaining in the well, and normalized to sham-irradiated control.

## Results

### Endogenous DNA-PKcs associates with PP6 and PP6R1 and Responds to Radiation

**Figure 1:**
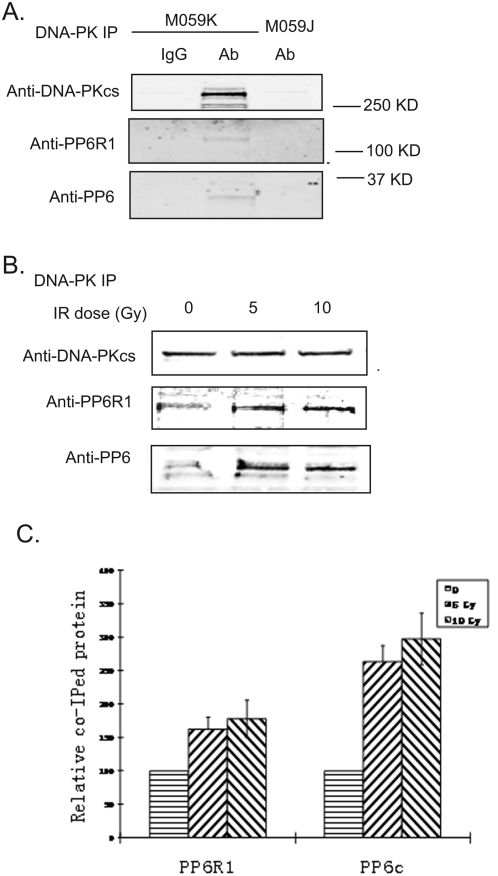
PP6R1 associates with DNA-PK in human cancer cells. A. Cell extracts from irradiated DNA-PK proficient (M059K), and deficient glioblastoma cells (M059J) were immunoprecipitated with monoclonal α-DNA-PKcs antibody. Following SDS-PAGE, DNA-PKcs, PP6R1 and PP6c proteins were detected by immunostaining using specific antibodies or pre-immune serum. B. M059K cells were irradiated with 5 or 10 Gy, or sham-treated (0 Gy). One hour after radiation, the cells were lysed and cytoplasmic and nuclei were prepared. Nuclei were subjected to immunoprecipitation with DNA-PKcs antisera. C. Immunoblots of the nuclear fractions were quantified by densitometry. Standard error bars represent the mean of three independent experiments (± SD). The statistical significance of the differences between the amount of PP6c or PP6R1 in the nuclear fraction from irradiated cells, and in the nuclear fraction from non-irradiated control cells was (***, p<0.001) by Student T test.

To investigate potential functions of PP6, we generated a stable 293 cell line expressing low levels of FLAG-tagged PP6R1 and analyzed immunoprecipitates by mass spectroscopy to identify interacting proteins [27]. This approach yielded multiple peptides from a total of >45 PP6R1 interacting proteins, which included PP6c, ankyrin-repeat PP6 subunits and DNA-PKcs. To follow-up on this observation, we prepared extracts from the glioblastoma cell lines M059K and M059J, which do or do not express DNA-PKcs, respectively, and immunoprecipitated with specific anti-DNA-PKcs antibodies. Immunoblotting showed endogenous PP6R1 and PP6c co-immunoprecipitated with endogenous DNA-PKcs from the proficient M059K cells using specific anti-DNA-PK antibodies, but not with non-immune IgG used as a negative control (Figure 1, lanes 1, 2). Using extracts from M059J cells neither PP6 nor PP6R1 were immunoprecipitated and, as expected, no DNA-PK was recovered (Figure 1, lane 3). These results establish that endogenous DNA-PKcs, PP6 and PP6R1 are associated in a complex that can be recovered by immunoprecipitation.

We tested whether the interaction of DNA-PKcs with PP6R1/PP6 was affected by IR. Endogenous DNA-PKcs was immunoprecipitated from nuclear extracts of M059K cells following various doses of radiation (0, 5, 10 Gy). Immunoblotting showed identical recovery of DNA-PKcs in each sample, but increased amounts of co-immunoprecipitated PP6R1 and PP6c from irradiated vs. non-irradiated cells (Figure 1B). Multiple independent experiments showed a statistically significant (1.6 to 2.6-fold) increase in co-precipitation of PP6R1 and PP6c following 5 Gy of IR (Figure 1C). There was little further increase in association with DNA-PKcs at 10 Gy compared to 5 Gy, and the relative- fold increase in amount of PP6R1 compared to PP6c when normalized to non-irradiated samples was slightly different, but the analyses involved immunoblotting with different antibodies for each subunit. These results showed that following IR there was a significant increase in the association of endogenous DNA-PKcs with PP6R1 and PP6c.

### DNA-PKcs is required for radiation-induced PP6R1 nuclear localization

**Figure 2:**
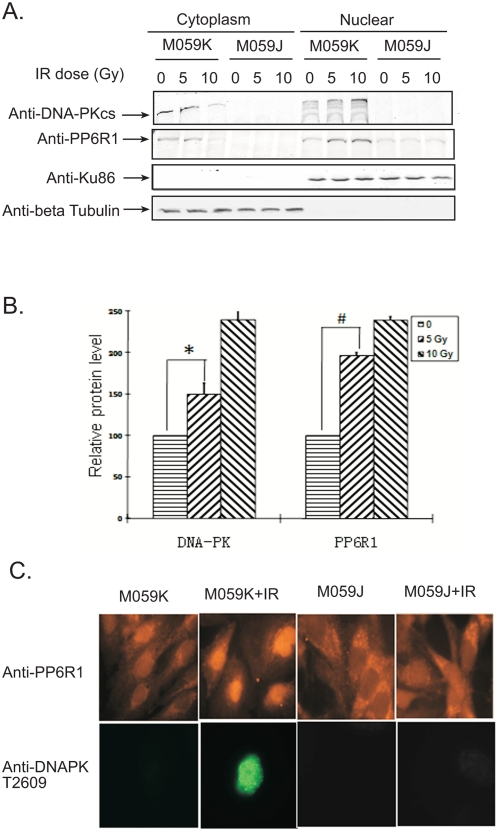
IR-induced nuclear localization of PP6R1 is DNA-PK dependent. A. M059K and M059J cells were left untreated or irradiated with 5 or 10 Gy. One hour after irradiation, cells were lysed and fractionated into cytoplasmic and nuclear fractions. The protein levels of DNA-PKcs and PP6R1 in these fractions were subjected to immunoblot analysis. β-Tubulin and Ku86 are markers for cytoplasmic and nuclear fractions, respectively. B. Immunoblots of the nuclear fractions were quantified by densitometry. The standard error bars represent means of three independent experiments (± SD). The statistical significance of the differences between the amount of DNA-PKcs or PP6R1 in the nuclear fraction from irradiated cells, compared with the nuclear fraction of non-irradiated control cells, was (***, p<0.001) by Student T test. C. M059K and M059J cells grown on chamber slides were irradiated with 5 Gy, or sham-irradiated. One hour post-irradiation, cells were fixed and immunostained with PP6R1 and DNA-PKcs antibodies. Upper row: orange PP6R1 staining, lower row: green phosphorylated DNA-PKcs Thr2609 stain.

DNA-PKcs is a central component of NHEJ that occurs in the nucleus. Therefore we asked if the IR –induced increase in the association of PP6R1with DNA-PKcs would affect the intracellular localization of endogenous PP6R1. We irradiated or mock-treated M059K and M059J glioblastoma cells, then prepared nuclear and cytoplasmic fractions that were analyzed by Western blotting (Figure 2A). After IR of either 5 or 10 Gy the levels of DNA-PKcs and PP6R1 were higher in the nucleus of M059K cells compared with non-irradiated (0 Gy) M059K cells. There was a corresponding decrease in the amount of DNA-PKcs and PP6R1 in the cytoplasmic fractions of irradiated vs. non-irradiated cells. The IR-induced increase in nuclear DNA-PKcs and PP6R1 was statistically significant at 5 Gy (Figure 2B) and the translocation from cytoplasm to nucleus was especially apparent (2.4-fold) with cells treated with 10 Gy compared to non-irradiated controls. Ku86 and tubulin were used as loading controls for the total amount of protein in the nuclear and cytoplasmic fractions, respectively (Figure 2A). As expected, DNA-PKcs was not detected by immunoblotting in the cytoplasm or nucleus of deficient M059J cells. The nuclear vs. cytoplasmic distribution of PP6R1 in DNA-PKcs deficient M059J cells was not affected by IR. These results showed that IR induced translocation of DNA-PKcs along with PP6R1 from the cytosol to the nucleus of glioblastoma cells.

Immunofluorescent microscopy was employed to visualize the intracellular localization of PP6R1 in response to IR. Endogenous PP6R1 was distributed in the cytoplasm and nucleus of M059K and M059J cells (Figure 2C, top row). Following IR there was increased staining of PP6R1 in the nucleus of M059K cells, but no similar increase in M059J cells. These results confirmed the cell fractionation analysis that showed IR-induced PP6R1 accumulation in the nucleus. We also observed an increase in staining for DNA-PKcs in the nucleus of irradiated M059K cells (data not shown), however, there was apparent cross-reactivity of the DNA-PKcs antibodies with other proteins that gave immunofluorescent staining of the cytoplasm of both M059K and M059J cells. Phosphosite-specific antibodies showed phosphorylation of Thr2609 in the nucleus of DNA-PKcs in irradiated but not control M059K cells. This staining was not seen following IR of M059J cells that are deficient in DNA-PKcs (Figure 2C). Taken together our results indicated that following IR there is an increased association of DNA-PKcs with PP6R1/PP6c and accumulation of the complex in the nucleus. The IR-induced nuclear localization of PP6R1 was seen in cells replete with DNA-PKcs (M059K), but not cells deficient in DNA-PKcs (M059J), suggesting that the kinase was required for relocalization of the phosphatase.

### Knockdown of DNA-PK abrogates radiation-induced PP6R1 nuclear localization

**Figure 3:**
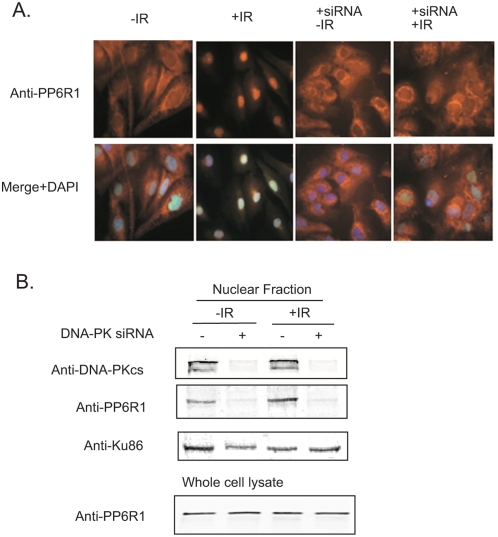
siRNA knockdown of DNA-PKcs abrogates IR-induced PP6R1 nuclear localization. A. M059K cells were transfected with control siRNA or anti-DNA-PKcs siRNA, and 48 hours later subjected to 5 Gy. One hour post-irradiation, the cells were fixed and immunostained with anti-DNA-PKcs and PP6R1 antibodies. Upper row: orange PP6R1 and lower row: merged Dapi ,DNA-PK and PP6R1. B. M059K cells were transfected with control siRNA or anti-DNA-PKcs siRNA and subjected to irradiation 48 hours post –transfection. One hour post-irradiation the cells were fractionated into cytoplasmic and nuclear fractions. The protein levels of DNA-PKcs, PP6R1, and Ku were detected by immunoblotting.

We tested the hypothesis that IR-induced nuclear localization of PP6R1 is dependent on DNA-PKcs. M059K cells were transfected with a pool of multiple siRNA to knockdown levels of DNA-PKcs or control siRNA. These cells were then irradiated or mock treated and compared for the distribution of endogenous PP6R1 by immunofluorescence microscopy (Figure 3A). In non-irradiated (-IR) cells transfected with control siRNA or specific siRNA the PP6R1 appeared predominantly in the cytoplasm compared to the nucleus, although we noted somewhat enhanced perinuclear staining and less nuclear staining for PP6R1 in the DNA-PK knockdown cells. Whereas M059K cells transfected with control siRNA showed greatly enhanced immunostaining for PP6R1 in the nucleus following IR, there was no IR-induced change in the distribution of PP6R1 in M059K cells knocked down for DNA-PK. As controls and for confirmation, nuclear extracts from parallel cultures were analyzed by immunoblotting (Figure 3B). The specific siRNA pool successfully depleted 90% of DNA-PK from the nuclei of M059K cells. The IR-induced accumulation of PP6R1 into the nucleus was evident in cells transfected with control siRNA, and there also was an IR-induced increase in nuclear DNA-PK, especially when normalized to Ku86 as loading control. Knockdown of DNA-PK (+lanes) essentially eliminated any accumulation of PP6R1 in the nucleus, in both irradiated and non-irradiated cells. One might speculate from this that DNA-PK is required for transport of the PP6 phosphatase complex into the nucleus, even in the absence of DNA damage. Ku86 was used as a loading control for the total amount of nuclear extract protein. Immunoblotting showed that the total amount of PP6R1 in these cells was unchanged by either siRNA transfection or IR, eliminating the possibility that increases or decreases in whole cell levels of endogenous PP6R1 accounted for the observed changes in distribution of the protein. Thus, there was cytoplasmic to nuclear redistribution of PP6R1 in response to IR that was dependent on expression of DNA-PKcs, supporting the observations made comparing M059K and M059J cells.

### Knockdown of PP6R1 or PP6c reduces activation of DNA-PK in response to IR

**Figure 4:**
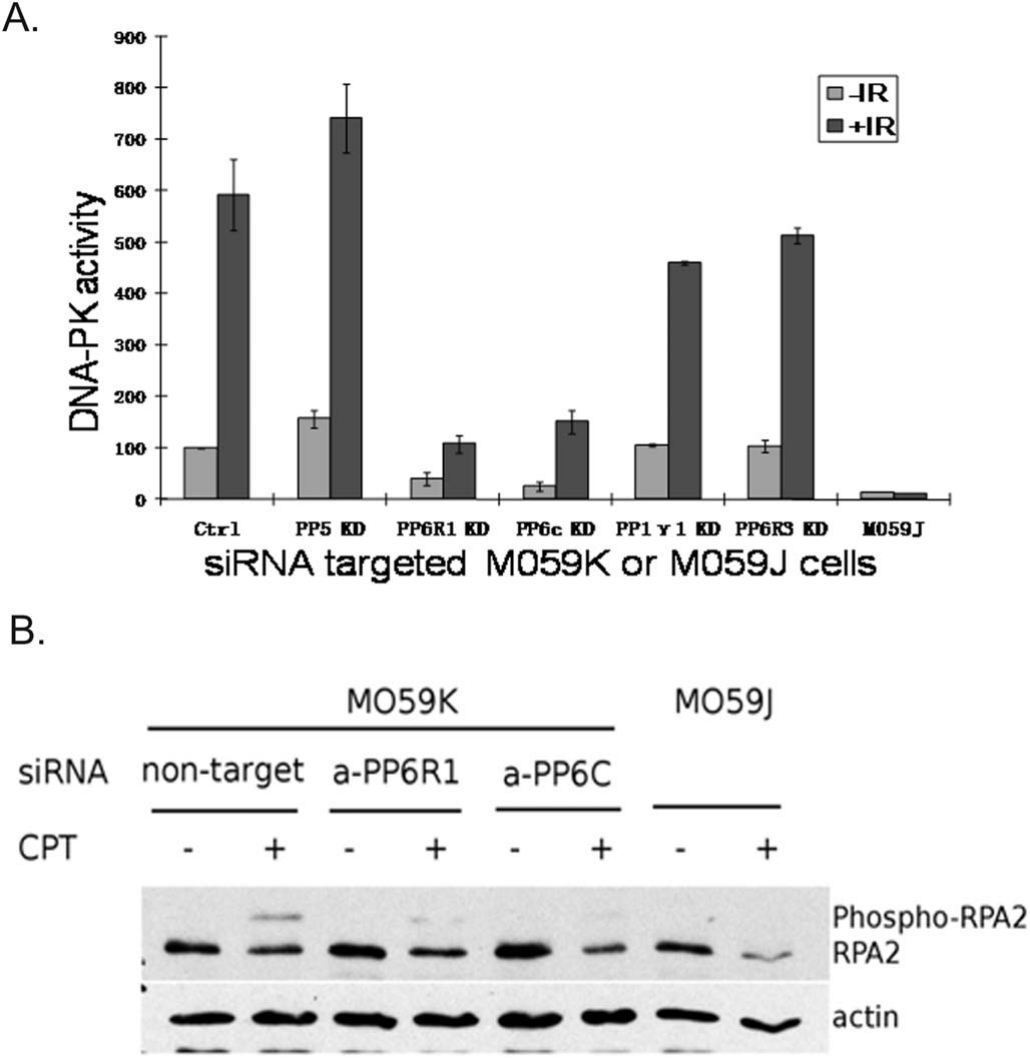
siRNA knockdown of PP6R1 significantly decreases DNA-PK kinase activity. M059K cells were transfected with control, non-targeting siRNA or specific siRNAs targeted to PP5, PP1γ1, PP6c, PP6R3 and PP6R1. A. Forty-eight hours after transfection, the M059K cells were irradiated with 5 Gy or sham-irradiated. Thirty minutes after irradiation, cells were harvested and fractionated. DNA-PKcs was immunoprecipitated from nuclear fractions, and the activity of DNA-PKcs was measured by incorporation of ^32^P into a DNA-PKcs-specific P53-derived peptide substrate. DNA-PKcs-deficient M059J cells were used as a negative control. The numbers were normalized to non-irradiated M059K cells and the data show the mean of counts per minute per µg protein in the eluted solution ± SD (n = 3). B. M059K cells transfected with anti-PP6c or anti-PP6R1 siRNA as described in panel A were treated with 1 µM CPT for 4 hours. RPA2 was detected in whole cell lysates by Western blot. . The slower migrating band represents the phosphorylated form of RPA2. M059J cells were used as a negative control.

DNA-PKcs contains multiple Ser/Thr phosphorylation sites, which are known to regulate DNA-PK catalytic and NHEJ activities. Does the association of PP6R1/PP6 with DNA-PK have a functional consequence, in terms of kinase activation in response to IR? To address this question M059K cells were transfected with pools of specific siRNA to knockdown individual phosphatases including PP6c, PP5 and PP1γ1, or the PP6 subunits PP6R1 or PP6R3. The activity of DNA-PKcs was analyzed in nuclear extracts using an *in vitro* kinase assay with a specific peptide substrate (Figure 4A). In this assay, DNA-PK activity in M059K cells transfected with control siRNA was increased about 6-fold in response to IR (black vs. grey bars). As a control to validate the assay, there was no detectable kinase activity in DNA-PK deficient M059J cells, either with or without IR. Knockdown of either PP6R1 or PP6c resulted in strong, almost complete, suppression of DNA-PK activity. There was residual IR-induced increase in DNA-PK in cells knocked down for PP6c or PP6R1, but this level of IR stimulated activity in the knockdown cells was about the same as for non-irradiated control cells (grey bars). The effects were highly selective for PP6 compared to other protein Ser/Thr phosphatases, even those with reported roles in control of DNA-PK. M059K cells knocked down for PP5 showed slightly elevated DNA-PK activity in response to IR, while knockdown of PP1γ1 allowed >4-fold kinase activation by IR, and a level of kinase slightly lower than control. These other Ser/Thr phosphatases, such as PP5 and PP1γ1, may be involved in the regulation of DNA-PK-mediated DNA repair, but did not directly regulate DNA-PKcs activity in this assay. Knockdown of another SAPS subunit for PP6 called PP6R3 did not significantly reduce IR-induced DNA-PK activity, attesting to the specificity for PP6/PP6R1. PP6R3 has a SAPS domain and like PP6R1 exclusively binds PP6 vs. other type-2A phosphatases. These data suggest that IR activation of DNA-PK requires the non-redundant action of a PP6 holoenzyme containing a PP6R1 subunit.

In addition, we tested whether PP6R1/PP6 was required for activation of DNA-PKcs in live cells, using the endogenous substrate replication protein A2 (RPA2, Figure 4B). RPA2 is differentially phosphorylated by three PI3Ks (ATM, ATR, DNA-PK) in response to different DNA damaging agents. However, DNA-PK is the primary kinase responsible for phosphorylating RPA2 in response to camptothecin (CPT) treatment [28]–[30]. Phosphorylation of RPA2 is seen in immunoblotting, by appearance of a band of reduced mobility relative to the RPA2 band. Camptothecin treatment induced phosphorylation of RPA2 in M059K cells, but not M059J cells. Knockdown of either PP6R1 or PP6 attenuated phosphorylation of the endogenous RPA2 in response to camptothecin, consistent with a PP6 requirement for activation of DNA-PK. We concluded that PP6/PP6R1 are required for activation of DNA-PK in response to two different DNA damaging agents (IR and CPT).

### DSB repair and glioblastoma survival after IR depend on PP6R1/PP6c

**Figure 5:**
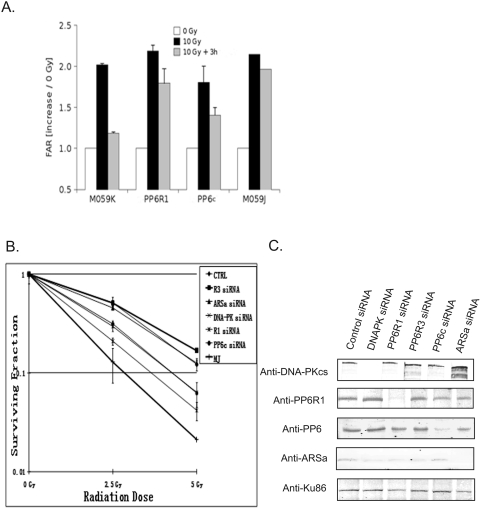
siRNA knockdown of PP6R1 or PP6c decreases repair of DNA double-strand breaks and sensitizes glioblastoma cells to radiation. A. Two days after transfection with siRNA cells were irradiated with 10 Gy and either harvested immediately (10 Gy) or allowed to repair DNA damage for 3 hours (10 Gy+3 hours) prior to harvesting cells. Harvested cells were embedded in agarose plugs and subjected to PFGE as described. The bars represent the fraction of DNA released from agarose plugs during PFGE, normalized to sham-irradiated control (±SEM). The data are from two independent experiments. M059J cells were used as a negative control. B. M059K cells were transfected with control siRNA or specific siRNAs targeted to DNA-PKcs, PP6R1, PP6c, PP6R3, or ARS-A. Two days after transfection, the M059K cells were irradiated with 0, 2.5, 5 Gy, replated, cultured for two weeks and scored for surviving colonies. The data points show the mean of surviving fraction ± SD (n = 3). M059J cells were used as a negative control. C. Representative Western blot for siRNA knockdown efficiency. The blot shows the level of the proteins of interest after transfection with siRNA. Ku86 is a marker for the nuclear fraction and was used as a loading control.

DNA-PK is central to NHEJ of DSB and because PP6R1/PP6 is required for IR-induced activation of DNA-PK, we wondered if DSB repair also was dependent on PP6R1 and PP6. We used pulse-field gel electrophoresis (PGFE) to measure DSB and repair. Glioblastoma M059K and M059J cells transfected with control siRNA were compared along with M059K cells transfected with siRNA pools targeted to PP6R1 or PP6c. Cells were irradiated with 10 Gy IR, and subjected to PFGE immediately, or 3 hr following radiation to allow for initial repair. In response to IR about the same level of DSB was detected in all four samples (black bars, Figure 5A). In the control M059K cells approximately 80% of DSB were repaired within 3 hr after radiation (grey bar), but this response was incomplete in PP6R1 or PP6c-depleted M059K cells, which show persistent DSB, as in the DNA-PK-deficient M059J cells. Thus, knockdown of either PP6R1 or PP6 mimics the deficiency in DSB repair seen in cells lacking DNA-PK itself, consistent with an impaired activation of DNA-PK kinase in response to IR.

Clonogenic survival is the ultimate *in vitro* assay for cellular responses to DNA damage. Cells are subjected to different doses of IR, then plated at limiting dilution and the number of individual colonies consisting of greater than 50 cells that grow out after a 10–14 days are scored and plotted as logarithm of the fraction of initial cells vs. radiation dose. M059K cells transfected with control siRNA and the DNA-PK deficient M059J cells were assayed to establish the range of response in the assay, with fewest colonies of M059J cells (lower line) and the most colonies of M059K cells (upper line) (Figure 5B). We transfected M059K cells with siRNA targeting DNA-PKcs, or PP6c, or the PP6 subunits PP6R1, PP6R3, ARS-A. Cells knocked down for PP6R1 or PP6c exhibited a survival rate similar to that of DNA-PKcs knockdown cells, separated above the line for M059J cells (Figure 5B). This response indicates a higher sensitivity to ionizing radiation. On the other hand, cells knocked down for the PP6 subunits PP6R3 or ARS-A were nearly the same as M059K cells transfected with control siRNA, showing no change in response to IR. Thus, there was a clear distinction in responses even between different subunits of PP6. Cell extracts were analyzed by immunoblotting for the level of the proteins of interest after siRNA transfection (Figure 5C). These results demonstrate the selectivity and effectiveness of the siRNA for the various targets, so different responses can be attributed to depletion of individual proteins. Ku86 was used as the loading control. We concluded that knock-down of PP6R1/PP6c enhanced radiation sensitivity of glioblastoma cells to nearly the same extent as a deficiency of DNA-PK itself.

## Discussion

Although the importance of DNA-PK in DNA repair is well established and has been studied extensively [1], [3], [5], [31], [32], the mechanism by which DNA-PK is regulated in response to IR remains unknown. The current model is that the ABCDE and PQR clusters in DNA-PKcs function reciprocally to regulate DNA end-access [3], [10], [15]. Phosphorylation of ABCDE promotes end-processing by increasing accessibility, whereas phosphorylation of PQR inhibits end-processing by decreasing accessibility. Phosphorylation-induced reduction of the protein kinase activity of DNA-PK is restored *in vitro* by the addition of the purified catalytic subunit of either PP1γ1 or PP2A and this reactivation is blocked by the potent protein phosphatase inhibitor microcystin [19]. Moreover, the Wabl group [18] reported that PP5 interacts with DNA-PK and that overexpression of PP5 changes the phosphorylation dynamics of two functional sites: Thr2609 and Ser2056. Another study [15] demonstrated that mutation to Glu to mimic phosphorylation at the conserved Thr3950 site gave an inactive the kinase, even though this mutation did not reduce the affinity of the catalytic subunit for DNA-bound Ku. These observations suggest that the phosphorylation status of DNA-PKcs could both positively and negatively modulate the DNA repair activity of DNA-PK.

Our data show that PP6R1/PP6 forms a complex with and activates DNA-PK in response to DNA damage. Knock-down of either PP6R1 or PP6c by siRNA significantly reduced the activity of DNA-PK in cells responding to IR. Thus, dephosphorylation by PP6, targeted by its PP6R1 subunit, offers a mechanism for the activation of DNA-PK protein kinase as part of the cellular damage response. Which sites in DNA-PKcs are the target of PP6 activity remain to be determined. Among the possibilities, phosphorylation of Thr2609 and Ser2056 was not affected by depletion of either PP6R1 or PP6c, using reagents available (data not shown). An inviting alternative is Thr3950, located in the kinase loop of DNA-PKcs as an inhibitory modification, however, phosphorylation of this site increased in response to IR [15]. PP6 may dephosphorylate sites in DNA-PKcs to reduce binding with heterodimer Ku proteins, because DNA-PK activation completely depends on Ku-mediated complex formation with DNA.

Our data show that endogenous DNA-PK and PP6R1 bind together, and radiation enhances their association and induces PP6R1 translocation into, or at least accumulation in, the nucleus. It is unclear what signal or modification, such as phosphorylation, triggers formation of the complex. PP6R1 does not have an obvious nuclear localization sequence. The association of PP6R1 with DNA-PK increases PP6R1 localization in the nucleus, possibly due to the nuclear import of the DNA-PK. Knock-down of DNA-PK prevents nuclear localization of PP6R1 and PP6, supporting the idea of a transport complex. Alternatively, nuclear DNA-PK could serve as an IR-activated anchor for localization of PP6R1/PP6 in the nucleus. That idea begs the question of how PP6 heterotrimers of Mr>450 kDa [27] enter the nucleus. Increased levels of the PP6R1/PP6/DNA-PK complex in the nucleus likely facilitate DNA repair and PP6 may have substrates other than DNA-PKcs. Interestingly, epidermal growth factor receptor (EGFR) was reported to interact with DNA-PKcs and to be over-expressed in tumors of epithelial origin [33], [34]. IR induces EGFR import into the nucleus, and inhibition of radiation-induced EGFR nuclear import by C225 (Cetuximab) suppressed DNA-PK activity [35]. Thus, EGFR may be involved in DNA PKcs activation, perhaps involving its kinase activity.

The subunit structure of PP6 has been described to include the catalytic subunit bound to a SAPS domain present in three different proteins, named PP6R1, PP6R2, PP6R3 [24]. The SAPS domain can alone bind PP6, and the SAPS domains in PP6R1, R2 and R3 closely resemble one another. It is possible more than one of the SAPS subunit might associate with DNA-PK, and this could in part account for the unequal co-precipitation of PP6R1 and PP6c with DNA-PK seen in Figure 1. However, siRNA knockdown of PP6R3 had little effect on DNA-PK activation or cell survival compared to PP6R1 or PP6c. The lack of antibodies for PP6R2 limits experiments to test these ideas. More recent evidence shows PP6 forms heterotrimers with the SAPS subunits, binding one of three different ARS [27]. This does not involve or require the SAPS domain but instead involves a C terminal region of these PP6 subunits. In this model the SAPS subunit act as a bridge or scaffold to simultaneously bind to separate domains the PP6c and the ARS subunit. It is proposed that the ankyrin repeats in the ARS are used to interact with substrates or may be involved in localization. Antibody reagents are only available for ARS-A, not the other proteins and knockdown of ARS-A by siRNA did not significantly affect clonogenic survival following IR. Therefore, ARS-A seems not required for PP6 effects on DNA-PK, and we imagine one of the other newly discovered ARS is involved in the complex with DNA-PK.

Our discovery that PP6 and PP6R1 associate with DNA-PK and are required for effective kinase activation in response to IR identify these proteins are possible drug targets for radiation sensitization. Small molecule selective inhibitors of PP6 are possible based on differences between PP6 and PP2A [21]. Alternatively, small molecule disruptors of PP6-PP6R1 or PP6R1-DNA-PK interaction are predicted to be selective in reduction of the repair response to IR and may be useful in radiotherapy.
